# Supplementary material for: Clinical characteristics and severity prediction score of Adenovirus pneumonia in immunocompetent adult
Source: PLoS One. 2023 Feb 16;18(2):e0281590. doi: 10.1371/journal.pone.0281590 (PMC9934457; doi:10.1371/journal.pone.0281590)
Supplement: Attachment — Submitted filename: PONE-D-17138 Pneumonia adenovirus immunocompetern sep 2022.docx [file pone.0281590.s002.docx]

**PONE-D-22-17138**

**CLINICAL CHARACTERISTICS AND SEVERITY PREDICTION OF ADENOVIRUS PNEUMONIA EIN IMMUNOCOMPETENT ADULTS**

This is a retrospective study during a 2-year period, in immunocompetent adults who develop pneumonia by Adenovirus. The authors found 50 patients and different prognostic scales of severity are used to know which one was more useful. They also describe main clinical and demographic characteristics, and found that SMART-COP score had the best association for severity.

**Main points**

Abstract

Lines 40-42 Adding all the co-infections (by bacterial, viruses and fungi), could be useful to find differences between ICU and non-ICU patients? It would be interesting to assess.

Methods

Lines 78-81. Include some characteristic of the hospital, such as the total number of hospital admissions per year, number of beds, and ICU.

In line 264 they wrote it is a regional respiratory center, put this also in the methods.

Lines 83-84. Do NGS or PCR are standardized to perform in all adults who are admitted because a pneumonia process? Please clarify.

lines 109-117. Also, searching for other pathogens, antibodies, bacterial cultures, are performed in all patients with low respiratory infections?

Results

Lines 133-134:…23 (46%) were admitted to or moved to the ICU. It should be interesting to know the number of patients who were admitted directly to the ICU, and how many moved later, including the mean time to admission to the ICU.

Lines 144-146. They describe the main results in the laboratory parameters. Should be interesting to comment something about monocytes, because included monocytopenia as a potential predictor in the background (line 59).

Line 148. The two patients who died the cause was directly related with the pneumonia? Another co-infection? septic shock? Please clarify.

Linea 150-151. The cost of hospitalization in ICU were higher. Including costs, and defining how they had calculated, must be included in the methods.

Line 169-170. The abbreviation next to the full name for Epstein-Barr must be first (exchange line 170 with 169). The common abbreviation is EBV, not EB, lines 170 and 277. EBV, should be included in the methods in the part of pathogen detection (lines 109-112)

Line. 170. Do IFA should be IFV? Or what does IFA mean?

Lines 173-175 and table 3. Add the whole gender and specie: Acinetobacter spp, Mycoplasma spp or pneumoniae? Streptococcus spp, Pseudomonas aeruginosa or spp?

Lines 179-187. The searching of lymphocyte subgroups should be added in the methods.

Figure 2 legend is very difficult to read. Describe also, the meaning of the different colors used.

**Minor**

I suggest the title should establish the severity prediction will be based in a previous score used for pneumonia patients. So maybe could be: “Clinical characteristics and severity prediction score of…….”

Introduction:

Lines 52-53: … with inadequate immunity, such as newborns and organ transplant recipients”. These infections are very common in hematopoietic stem cell transplants. I suggest to add these too.

Methods

line 113. Describe the abbreviations for IFA, or it should be IFV?

Results

Line 134. Approximately 89.96% (20/23) of patients who required ICU…. Approximately? Consider to eliminate the word

Eliminate the two decimals (.00) and leave the whole number

Tables

IN the first column, you can leave the number, example (n=23, %). And eliminate all the symbol % in all the rest of the lines.

Typo

Line 235. Should be pathogens, nor parthenogens
