# Supplementary material for: Clinical characteristics and severity prediction score of Adenovirus pneumonia in immunocompetent adult
Source: PLoS One. 2023 Feb 16;18(2):e0281590. doi: 10.1371/journal.pone.0281590 (PMC9934457; doi:10.1371/journal.pone.0281590)
Supplement: Attachment — Submitted filename: renamed_9d910.docx [file pone.0281590.s003.docx]

**Response to Reviewers' comments**

**Reviewer #1**

***Comment 1:*** *thank you for the opportunity to review this manuscript
after reading it there are several doubts and questions that arise.
1) a study period comprising the first year of the covid pandemic was chosen: are the periods before covid and during covid comparable? was the hospital management similar? were the diagnostic studies performed the same? were there no changes that would make the two time periods not comparable?*

**Response:** Thanks for reviewer’s comments.

Undoubtedly, the pandemic has affected human's lifestyle, such as wearing masks and nucleic acid testing. Our study spanned the pandemic period from 2018 to 2020, but the outbreak of adenovirus pneumonia was mainly prevalent in 2019, similar to the study from XU et al [1]. There were only four cases in 2018 and 2020.

The admission time of the enrolled patients is shown below：

|  | 人数 |
| --- | --- |
| 2018.02 | 1 |
| 2018.04 | 1 |
| 2019.03 | 6 |
| 2019.04 | 11 |
| 2019.05 | 11 |
| 2019.06 | 9 |
| 2019.07 | 4 |
| 2019.08 | 2 |
| 2019.12 | 3 |
| 2020.08 | 1 |
| 2020.10 | 1 |

Due to government policy for the pandemic, there were only 36 cases confirmed COVID-19 in Xiangtan city from January 24, 2020 to March 5, 2020, and no new cases in this city until November 2020. All 36 confirmed cases were managed in a closed-loop hospital (a linear distance of 3.1 KM away from our regional respiratory center).

Since 2020, patients admitted to our hospital was required to provide a negative SARS-CoV-2 nucleic acid test during 48 hours and green health code [2]. The hospital management did not affect our diagnostic procedures for adenovirus pneumonia. This study was aim to explore the clinical features of adenovirus pneumonia in immunocompetent population and to validate the optimal ICU admission severity score. In view of the above, we believe that the covid pandemic did not influence the conclusion in our study.

1. Xu D, Chen L, Wu X, Ji L. Molecular typing and epidemiology profiles of human adenovirus infection among hospitalized patients with severe acute respiratory infection in Huzhou, China. PLoS One. 2022 Apr 21;17(4):e0265987. doi: 10.1371/journal.pone.0265987. PMID: 35446868; PMCID: PMC9022850.
2. Dong J, Wu H, Zhou D, Li K, Zhang Y, Ji H, Tong Z, Lou S, Liu Z. Application of Big Data and Artificial Intelligence in COVID-19 Prevention, Diagnosis, Treatment and Management Decisions in China. J Med Syst. 2021 Jul 24;45(9):84. doi: 10.1007/s10916-021-01757-0. PMID: 34302549; PMCID: PMC8308073.

***Comment 2:*** *The use of the term "immunocompetent adults" is strongly emphasized, considering that there are diabetics (whose level of metabolic control is unknown) and also patients with chronic obstructive pulmonary disease (COP).*

**Response:** Thanks for reviewer’s comments, we have revised in Method part as” Given patients with common comorbid conditions such as diabetes, chronic lung disease, or even those who are pregnancy are typically infected with the same spectrum of organisms that cause CAP in healthier adults, our study did not exclude these patients.” in line 83-96.

***Comment 3:*** *in one of the tables it is mentioned that a significant number of patients have use of sterioids (26 patients), it is not clear if they were used as part of the treatment of pneumonia or were drugs used prior to admission.*

**Response:** Thanks for reviewer’s comments, we have revised in Result part as” 26 patients (52%) used corticosteroid as part of the treatment of pneumonia. “in line 165-166.

***Comment 4:*** *also in the definition of immunocompetent, was HIV co-infection studied on admission of the patients? no mention is made of whether this was ruled out.*

*You must explain the meaning of an acronym before using it.*

**Response:** Thanks for reviewer’s comments, we have revised in Method part as” Patients were not allowed to participate in the trial if they possessed any of the subsequent: a) Immunocompromised; b) No acute lower respiratory symptoms; c) No pneumonia signs on chest radiography or computed tomography (CT); (Fig 1). Immunocompromised patients in CAP were defined as below: (i) Human immunodeficiency virus infection with CD4+ lymphocyte count < 200 cells/μL or percentage ＜14% or by the occurrence of an AIDS-defining condition; (ii) hematopoietic stem cell and solid organ transplantation; (iii) receiving chemotherapy prescribed for ≥ 3 months before admission; (iv) receiving corticosteroid use (≥20 mg/day of prednisone for ≥14d or＞600mg cumulative dose of prednisone); (v) receiving biological immunotherapy during the last 6 months;(vi) Receiving disease-modify antirheumatic drugs or other immunosuppressive drugs (eg, cyclophosphamide, methotrexate, cyclosporin, hydroxychloroquine); (vii) congenital/genetic immunodeficiencies; (viii)active malignancy or malignancy during 1 year. Given patients with common comorbid conditions such as diabetes, chronic lung disease, or even those who are pregnancy are typically infected with the same spectrum of organisms that cause CAP in healthier adults, our study did not exclude these patients.” in line 83-96.

***Comment 5:*** *The high rate of infection with agents that are not part of the usual community pneumonias is striking. 34% of infection with Acinetobacter spp, also Tuberculosis, even fungal pneumonias (aspergillus which exists almost exclusively in immunocompromised patients). How can this rate of infection be explained?*

**Response:** Thanks for reviewer’s comments. In our study, co-infection with Acinetobacter occurred in 8 cases in 23 ICU patients and only 1 case in 27 non-ICU patients. The average time of admission to the ICU was 0.875 days, and the mean ICU stay was 9.25 days for these eight patients. Admission to ICU ≤48 hours and long ICU stays were risk factors for secondary infection with Acinetobacter after viral infection [1]. China is a high incidence of Tuberculosis, accounting for 8.5% of new TB cases globally in 2020, with 842,000 new occurrences [2]. Meanwhile, Acinetobacter and TB have also been reported in CAP infections in immunocompetent populations [3]. A retrospective cohort study from six academic hospitals in China found Aspergillus was commonly co-infected with viral CAP in the immunocompetent group [4]. Our study's two patients infected with Aspergillus did not have any underlying disease, and Aspergillus was obtained by culture in alveolar lavage fluid. In addition, we found that the lymphocyte subset was decreased in adenovirus pneumonia, which may increase the potential infectious opportunity of these pathogens.

1. Ripa M, Galli L, Poli A, et al. Secondary infections in patients hospitalized with COVID-19: incidence and predictive factors. Clin Microbiol Infect. 2021 Mar;27(3):451-457. doi: 10.1016/j.cmi.2020.10.021.
2. <https://www.who.int/publications/i/item/9789240037021>
3. Carugati M, Aliberti S, Sotgiu G, et al. Bacterial etiology of community-acquired pneumonia in immunocompetent hospitalized patients and appropriateness of empirical treatment recommendations: an international point-prevalence study. Eur J Clin Microbiol Infect Dis. 2020 Aug;39(8):1513-1525. doi: 10.1007/s10096-020-03870-3.
4. Li L, Hsu SH, Wang C, et al. Characteristics of viral pneumonia in non-HIV immunocompromised and immunocompetent patients: a retrospective cohort study. BMC Infect Dis. 2021 Aug 6;21(1):767. doi: 10.1186/s12879-021-06437-5.

**Reviewer #2**

***Comment 1:*** *Main points
Abstract
Lines 40-42 Adding all the co-infections (by bacterial, viruses and fungi), could be useful to find differences between ICU and non-ICU patients? It would be interesting to assess.*

**Response:** Thanks for reviewer’s comments, we have revised in Method part as” Coinfection with virus was more common in non-ICU patients than ICU patients (13[48.15%]VS 4[17.39%],P=0.024), while bacteria and fungi not.” in line 38-40.

***Comment 2:*** *Methods
Lines 78-81. Include some characteristic of the hospital, such as the total number of hospital admissions per year, number of beds, and ICU.*

**Response:** Thanks for reviewer’s comments, we have revised in Method part as” The research was done at Xiangtan Central Hospital, a tertiary teaching hospital with 2800 beds and 88,694 hospital admissions per year, with approval by Xiangtan Central Hospital Medical Ethics Committee (NO.2020-11-003).” in line 76-78.

***Comment 3:*** *In line 264 they wrote it is a regional respiratory center, put this also in the methods.*

**Response:** Thanks for reviewer’s comments, we have revised in Discussion part as” Second, as a regional respiratory center with 14 beds and 200 ICU admissions per year,” in line 308.

***Comment 4:*** *Lines 83-84. Do NGS or PCR are standardized to perform in all adults who are admitted because a pneumonia process? Please clarify.*

**Response:** Thanks for reviewer’s comments, we have revised in Method part as” NGS and PCR test were standardized performed at BGI Clinical Laboratories (Shenzhen) Co., Ltd and Sansure(Changsha) Biotech Inc, respectively.” in line 82-84.

***Comment 5:*** *lines 109-117. Also, searching for other pathogens, antibodies, bacterial cultures, are performed in all patients with low respiratory infections?*

**Response:** Thanks for reviewer’s comments, we have revised in Method part as” Coinfections were tested in all enrolled patients and determined positive if they met one of the following:” in line 123-124.

***Comment 6:*** *Results
Lines 133-134:…23 (46%) were admitted to or moved to the ICU. It should be interesting to know the number of patients who were admitted directly to the ICU, and how many moved later, including the mean time to admission to the ICU.*

**Response:** Thanks for reviewer’s comments, we have revised in Result part as” The mean time to admission to the ICU was 1.04 day with 15 patients admitted directly to the ICU and 5 patients moved later.” in line 150-152.

***Comment7:*** *Lines 144-146. They describe the main results in the laboratory parameters. Should be interesting to comment something about monocytes, because included monocytopenia as a potential predictor in the background (line 59).*

**Response:** Thanks for reviewer’s comments, we have revised in Result part as” Monocytes was lower in ICU patients than non-ICU patients though no differ significantly.” in line 163.

***Comment 8:*** *Line 148. The two patients who died the cause was directly related with the pneumonia? Another co-infection? septic shock? Please clarify.*

**Response:** Thanks for reviewer’s comments, we have revised in Result part as” two died both for severe pneumonia.” in line 168.

***Comment 9:*** *Linea 150-151. The cost of hospitalization in ICU were higher. Including costs, and defining how they had calculated, must be included in the methods.*

**Response:** Thanks for reviewer’s comments, we have revised in Methods part as” Hospitalization stays and cost were collected. Hospitalization expenses include bed fee, medical service fee (doctor's consultation fee, nursing fee), examination fee (CT, B-ultrasound, hematology examination, etc.), drug fee, rehabilitation fee, and the cost of materials used during hospitalization (such as pipes, needles, etc.).” in line 107-109.

***Comment 10:*** *Line 169-170. The abbreviation next to the full name for Epstein-Barr must be first (exchange line 170 with 169). The common abbreviation is EBV, not EB, lines 170 and 277. EBV, should be included in the methods in the part of pathogen detection (lines 109-112)*

**Response:** We agree with the Reviewer. It was revised.

***Comment 11:*** *Line. 170.Do IFA should be IFV? Or what does IFA mean?*

**Response:** Thanks for reviewer’s comments. We apologize for the mistake that occurred during manuscript translation and preparation. we have revised in manuscript.

***Comment 12:*** Lines 173-175 and table 3. Add the whole gender and specie: Acinetobacter spp, Mycoplasma spp or pneumoniae? Streptococcus spp, Pseudomonas aeruginosa or spp?

**Response:** Thanks for reviewer’s comments, we have revised in manuscript.

***Comment 13:*** *Lines 179-187. The searching of lymphocyte subgroups should be added in the methods.*

**Response:** Thanks for reviewer’s comments, we have revised in Methods part as”” in line 122 and line 132-133.

***Comment 14:*** *Figure 2 legend is very difficult to read. Describe also, the meaning of the different colors used.*

**Response:** Thanks for reviewer’s comments, we have revised in Result part as” Percentage in Fig means percentage of patients tested value beyond normal value in all tested patients except CD3-CD19+ (%) (percentage of lower-than-normal value) and CD3+/CD4+ (percentage in the range of normal value reference). Normal value references:CD3-CD16+CD56+(#),150-1100M/L; CD3-CD19+(#),90-560M/L; CD3+CD19-(#),955-2860M/L; CD3+CD4+(#),550-1440M/L; CD3+CD8+(#),320-1250M/L; CD3+CD19-(%),56-84%; CD3+CD4+(%),27-51%; CD3+CD8+(%),15-44%; CD3-CD16+CD56+(%),7-40%; CD3-CD19+(%), 5-18%. * p = 0.046.” in line 218-225.

***Comment 15:*** *Minor
I suggest the title should establish the severity prediction will be based in a previous score used for pneumonia patients. So maybe could be: “Clinical characteristics and severity prediction score of…….”*

**Response:** We agree with the Reviewer. It was revised.

***Comment 16:*** *Introduction:
Lines 52-53: … with inadequate immunity, such as newborns and organ transplant recipients”. These infections are very common in hematopoietic stem cell transplants. I suggest to add these too.*

**Response:** We agree with the Reviewer. It was revised.

***Comment 17:*** *Methods
line 113. Describe the abbreviations for IFA, or it should be IFV?*

**Response:** Thanks for reviewer’s comments, we have revised in manuscript.

***Comment 18:*** *Line 134. Approximately 89.96% (20/23) of patients who required ICU…. Approximately? Consider to eliminate the word*

**Response:** Thanks for reviewer’s comments, we have revised in manuscript.

***Comment 19:*** *Eliminate the two decimals (.00) and leave the whole number*

**Response:** Thanks for reviewer’s comments, we have revised in manuscript.

***Comment 20:*** *Tables
IN the first column, you can leave the number, example (n=23, %). And eliminate all the symbol % in all the rest of the lines.*

**Response:** Thanks for reviewer’s comments, we have revised in Tables part.

***Comment 21:*** *Typo
Line 235. Should be pathogens, nor parthenogens*

**Response:** Thanks for reviewer’s comments, we have revised in manuscript.

**Reviewer #3**

***Comment 1:*** *Abstract should have the sample size used for the study.*

**Response:** Thanks for reviewer’s comments, we have revised in Abstract part as” We retrospectively reviewed 50 Adenovirus pneumonia inpatients in Xiangtan Central Hospital from 2018 to 2020.” in line 24-25.

***Comment 2:*** *End of the manuscript (line 262) mentioned in there that 56 patients, this needs to be clarified in the methodological section appropriately.*

**Response:** Thanks for reviewer’s comments, we have revised in Discussion part as” there were only 50 patients eligible for the study.” in line 306.

***Comment 3:*** *Methodological details regarding RT-PCR or NGS should be given - at least some minimal details need to be given.*

**Response:** Thanks for reviewer’s comments, we have revised in Method part as” NGS and PCR test were standardized performed at BGI Clinical Laboratories (Shenzhen) Co., Ltd and Sansure(Changsha) Biotech Inc, respectively.” in line 80-82.

***Comment 4:*** *When the manuscript focuses immunocompetent individuals, including some diseased/clinical conditions such as Diabetes and Pregnancy may not be appropriate.*

**Response:** Thanks for reviewer’s comments, we have revised in Methods part as” Patients were not allowed to participate in the trial if they possessed any of the subsequent: a) Immunocompromised; b) No acute lower respiratory symptoms; c)No pneumonia signs on chest radiography or computed tomography (CT); (Fig 1). Immunocompromised patients in CAP were defined as below[14-15]: (i) Human immunodeficiency virus infection with CD4+ lymphocyte count < 200 cells/μL or percentage ＜14% or by the occurrence of an AIDS-defining condition; (ii) hematopoietic stem cell and solid organ transplantation; (iii) receiving chemotherapy prescribed for ≥ 3 months before admission; (iv) receiving corticosteroid use (≥20 mg/day of prednisone for ≥14d or＞600mg cumulative dose of prednisone); (v) receiving biological immunotherapy during the last 6 months;(vi) Receiving disease-modify antirheumatic drugs or other immunosuppressive drugs (eg, cyclophosphamide, methotrexate, cyclosporin, hydroxychloroquine); (vii) congenital/genetic immunodeficiencies; (viii)active malignancy or malignancy during 1 year. Given patients with common comorbid conditions such as diabetes, chronic lung disease, or even those who are pregnancy are typically infected with the same spectrum of organisms that cause CAP in healthier adults [14], our study did not exclude these patients.” in line 83-96.

***Comment 5:*** *Table 3: All the bacteria terms should be initialized.*

**Response:** Thanks for reviewer’s comments, we have revised in Table 3.

**Editor**

***Comment 1:*** *Please review comments made by the reviewers and provide point by point response in your revised manuscript. Specifically please address study methodology and criteria used to define immunocompetent adults.*

**Response:** Thanks for your comments, we have revised in Manuscript.

***Comment 2:*** *When submitting your revision, we need you to address these additional requirements.1. Please ensure that your manuscript meets PLOS ONE's style requirements, including those for file naming. The PLOS ONE style templates can be found at https://journals.plos.org/plosone/s/file?id=wjVg/PLOSOne_formatting_sample_main_body.pdf and*

[*https://journals.plos.org/plosone/s/file?id=ba62/PLOSOne_formatting_sample_title_authors_affiliations.pdf*](https://journals.plos.org/plosone/s/file?id=ba62/PLOSOne_formatting_sample_title_authors_affiliations.pdf)*.*

**Response:** We revised the sections accordingly.

***Comment 3:*** *Thank you for stating the following in the Acknowledgments Section of your manuscript:“This work was supported by the Medical scientific research project in Xiangtan city [grant number 2020xtyx-6]”We note that you have provided funding information that is not currently declared in your Funding Statement. However, funding information should not appear in the Acknowledgments section or other areas of your manuscript. We will only publish funding information present in the Funding Statement section of the online submission form.Please remove any funding-related text from the manuscript and let us know how you would like to update your Funding Statement. Currently, your Funding Statement reads as follows:“The funders had no role in study design, data collection and analysis, decision to publish, or preparation of the manuscript.”Please include your amended statements within your cover letter; we will change the online submission form on your behalf.We note that the grant information you provided in the ‘Funding Information’ and ‘Financial Disclosure’ sections do not match. When you resubmit, please ensure that you provide the correct grant numbers for the awards you received for your study in the ‘Funding Information’ section.*

**Response:** We revised the sections accordingly.

***Comment 4:*** *Please remove your figures from within your manuscript file, leaving only the individual TIFF/EPS image files, uploaded separately.  These will be automatically included in the reviewers’ PDF.*

**Response:** We revised the sections accordingly.
