# Supplementary material for: Clinical characteristics and severity prediction score of Adenovirus pneumonia in immunocompetent adult
Source: PLoS One. 2023 Feb 16;18(2):e0281590. doi: 10.1371/journal.pone.0281590 (PMC9934457; doi:10.1371/journal.pone.0281590)
Supplement: Attachment — Submitted filename: renamed_77903.docx [file pone.0281590.s004.docx]

**Response to Reviewers' comments**

Dear Editors and Reviewers:

Thank you for your letter and for the reviewers’ comments concerning our manuscript. Those comments are all valuable and very helpful for revising and improving our paper, as well as the important guiding significance to our researches. We have studied comments carefully and have made correction which we hope meet with approval. The main corrections in the paper and the responds to the reviewers’ comments are as following.

We appreciate for Editors and Reviewers’ warm work earnestly, and hope that the correction will meet with approval.

Once again, thank you very much for your comments and suggestions.

Best regards,

**Ming Yan Jiang**

**Reviewer #1**

***Comment 1:*** *Given the high rate of co-infection with other agents, it is very difficult to assert that a score can predict the severity of adenovirus pneumonia when in reality it is mostly co-infection with multiple agents.
In the conclusions it is stated: "In conclusion, adenovirus pneumonia is not uncommon in immunocompetent adult patients who are prone to coinfection with multiple agents."
This part of the conclusion, in my opinion, if it is possible to extract from the study done.
However, the second part of the conclusion: "Our data suggest that the initial SMART-COP score is a valid predictive assessment of ICU admission in adult non-immunocompromised patients hospitalized with adenovirus pneumonia" does not seem to me to be drawn from the reported study.*

**Response:** Thanks for reviewer’s comments. To further explore influence of co-infection on SMART-COP, we divided enrolled patients into two groups (co-infections and no co-infections) and found that the distribution of SAMRT-COP score in two groups was similar with no statistical difference. Therefore, we thought that co-infection does not influence SMART-COP score in predicting ICU admission risk for adenovirus pneumonia.

In addition, given that our study was a retrospective single-center investigation, we will conduct prospective, multicenter studies in the future to explore the impact of co-infection on the predictive score of viral CAP.

| **SMART-COP** | ICU care | No ICU care |
| --- | --- | --- |
| **Co-infections** |  |  |
| Low risk (0-2 points) | 0 | 12 |
| Moderate risk (3-4 points) | 13 | 7 |
| High risk (5-6 points) | 3 | 0 |
| Very high risk (≥7 points) | 1 | 0 |
| **No Co-infections** |  |  |
| Low risk (0-2 points) | 0 | 2 |
| Moderate risk (3-4 points) | 5 | 6 |
| High risk (5-6 points) | 1 | 0 |
| Very high risk (≥7 points) | 0 | 0 |

Therefore, we thought that co-infection does not influence SMART-COP score in predicting ICU admission risk for adenovirus pneumonia.

In addition, given that our study was a retrospective single-center investigation, we will conduct prospective, multicenter studies in the future to confirm the impact of co-infection on the predictive score of viral CAP.

**Reviewer #2**

***Comment 1:*** *All the suggestions were made.
It is only important to correct the names of the bacteria: if it has a gender and specie, it is no longer necessary to include spp. (eg. Klebsiella pneumoniae). If it is only the genus without species, must include spp (species), example: Acinetobacter spp. Run lines 204, 205, table 3, and everything that corresponds*

**Response:** Thanks for reviewer’s comments, we have revised in Line 204-206 and Table 3.
