# Supplementary material for: Clinical characteristics and severity prediction score of Adenovirus pneumonia in immunocompetent adult
Source: PLoS One. 2023 Feb 16;18(2):e0281590. doi: 10.1371/journal.pone.0281590 (PMC9934457; doi:10.1371/journal.pone.0281590)
Supplement: Attachment — Submitted filename: renamed_95dea.docx [file pone.0281590.s005.docx]

**Response to Reviewers' comments**

Dear Editors and Reviewers:

Thank you for your letter and for the reviewers’ comments concerning our manuscript. Those comments are very valuable and very helpful for revising and enhancing our work, as well as providing crucial guidance for our study. We carefully reviewed the concerns and made changes that we hope will meet with approval. The following are the responses to the reviewers' remarks.

We sincerely thank the Editors and Reviewers for their hard work and hope that the correction will meet with approval.

Thank you for your comments and suggestions once more.

Best regards,

**Ming Yan Jiang**

**Reviewer #1**

***Comment 1:*** *in my opinion the separate results for co-infected versus non co-infected should be included and highlight that there was no significant difference, implying that co-infections do not affect the validity of the SMART-COP score.*

**Response:** Thanks for reviewer’s comments, we have revised in Manuscript.
